# Pediatric Thoracic Injury Rule out Criteria (pTIRC) in Diagnosis of Very Low Risk Children for Traumatic Intrathoracic Injuries; a Diagnostic Accuracy Study

**Published:** 2020-01-08

**Authors:** Mahmoud Yousefifard, Mostafa Hosseini, Mohammad Reza Parvizi

**Affiliations:** 1 Department of Medicine, AJA University of Medical Science Tehran, Iran.; 2 Pediatric Chronic Kidney Disease Research Center, Tehran University of Medical Sciences, Tehran, Iran.; 3 Department of Physiology, School of Medicine, AJA University of Medical Science Tehran, Iran.

**Keywords:** Clinical decision rules, reproducibility of results, multiple trauma, sensitivity and specificity

## Abstract

**Introduction:**

The value of thoracic injury rule out criteria (TIRC) as a tool for decreasing the number of unnecessary chest radiographs in children has not been evaluated yet. Therefore, the present study was designed as a multi-center study to assess the validity of TIRC model in detection of very low risk children for traumatic intrathoracic injuries.

**Methods:**

In this diagnostic accuracy study, clinical data and chest radiographs of 974 children less than 18 years of age (72.0% boys) who had presented to 5 hospitals, in Iran in 2018 were assessed. Data gathering and interpretation of radiographs were done by two independent researchers in each hospital. In the end, discriminatory power and calibration of the model was assessed with a 95% confidence interval (95% Cl).

**Results:**

In the present study, age was not a predicting factor of abnormal findings in radiographs of children and adolescents (p=0.75); therefore, it was omitted from TIRC model and pediatric TIRC (pTIRC) was designed. Area under the curve of pTIRC rule was 0.97 (95% CI: 0.96-0.98) for prediction of abnormal chest X-Ray in children and adolescents. The sensitivity and specificity of pTIRC was 100% and 90.1%, respectively. The calibration of this decision rule had great concordance with the perfect line with a slope of 0.99 and intercept of 0.001. There was a 90.1% reduction in the number of unnecessary chest radiographs when using pTIRC decision rule.

**Conclusion:**

pTIRC decision rule was introduced in the present study. pTIRC has excellent performance in identification of traumatic intrathoracic injuries and decreasing the number of unnecessary chest radiographs.

## Introduction:

According to advanced trauma life support guidelines, a chest X-Ray (CXR) is necessary for all patients presenting to health care centers with multiple trauma in order to rule out traumatic intrathoracic injuries (1). However, studies show that chest radiographs are unnecessary in many circumstances and also low diagnostic yield is reported for CXR (2-5). Therefore, decision rules were designed to reduce the number of CXRs in patients with multiple trauma (6, 7).

NEXUS chest was the first decision rule designed for identification of clinically important thoracic injuries following chest trauma. In this model, age more than 60 years, rapid deceleration, presence of chest pain, chest wall tenderness, distracting painful injury, altered mental status and intoxication were considered the most important factors in identification of high risk patients for presence of intrathoracic injuries following blunt chest trauma. However, speed of vehicle at the time of accident being a required factor was a limitation of this model as such data are not usually accessible (6).

Therefore, Forouzanfar et al. designed and introduced thoracic injury rule out criteria (TIRC). This model, which is based on data gathered from adults, indicates that a patient who is hemodynamically stable without loss of consciousness and is under 60 years old with no wall pain, chest wall tenderness, decrease in pulmonary sound, crepitation, chest skin abrasion, or dyspnea is considered very low risk and does not require a chest radiograph (6). 

Although both NEXUS chest and TIRC models have been validated in adults, there is a wide gap in the field of pediatrics. Since calculations of TIRC decision rule is easier than NEXUS chest and its variables can be assessed more readily in the clinic, the present study assesses the validity of TIRC in decreasing the number of unnecessary chest radiographs in children with multiple trauma for the first time.

## Methods:

***Study design and setting***

In the present diagnostic accuracy study, the value of TIRC rule in decreasing the number of unnecessary radiographs was assessed in children with multiple traumas presenting to 5 hospitals in 2018. The study protocol was approved by ethics committee of Aja University of Medical Sciences (code: 91000283). Throughout the study period, all researchers adhered to the principles of the Helsinki declaration and a consent form was signed by patients or their parents. In addition, data gathering was performed by a physician not included in the medical team of the patient.

***Participants***

Children under the age of 18 years presenting to emergency department were included using convenience sampling. Exclusion criteria were penetrating trauma, lack of consent, lack of a chest radiograph due to decision of the patient’s physician, patients with a chest radiograph before clinical assessment and discharge before completing data gathering.

***Data gathering***

Patients were assessed in a prospective manner from admission and related data were gathered in a predesigned form. Assessed data included age, sex, mechanism of trauma, level of consciousness, heart rate (HR), systolic blood pressure (SBP), diastolic blood pressure (DBP), respiratory rate (RR), oxygen saturation level (SaO2), and presence of dyspnea, distracting pain, chest skin abrasion, chest tenderness, chest deformity, crepitation, decreased pulmonary sound, chest wall pain, and subcutaneous emphysema. Selecting these factors was based on the study by Forouzanfar et al. (6).

**Table 1:** Baseline and clinical characteristics of studied children based on chest X ray (CXR) findings

**Variable**	**CXR Findings**		**Total** **(n=974)**	**P value**
	**Normal (n=848)**	**Abnormal (n=126)**		
**Age (year)**				
1-3	123 (14.5)	14 (11.1)	137 (14.1)	0.75
4-6	182 (21.5)	30 (23.8)	212 (21.8)	
7-12	352 (41.5)	54 (42.9)	406 (41.7)	
13-17	191 (22.5)	28 (22.2)	219 (22.5)	
**Gender **				
Boys	622 (73.4)	79 (62.7)	701 (72.0)	0.01
Girls	226 (26.6)	47 (37.3)	273 (28.0)	
**Mechanism of injury **				
Motorcycle accident	112 (13.3)	20 (15.9)	132 (13.6)	0.007
Car accident	231 (27.3)	47 (37.3)	278 (28.7)	
Pedestrian accident	338 (40.0)	39 (31.0)	377 (38.9)	
Fall ≥ 1.5 meter	40 (4.7)	10 (7.9)	50 (5.2)	
Fall < 1.5 meter	77 (9.1)	10 (7.9)	87 (9.0)	
Other	46 (5.0)	0 (0.0)	46 (4.7)	
**GCS **				
15	790 (93.2)	77 (61.1)	867 (89.0)	<0.0001
13-14	11 (1.3)	29 (23.0)	40 (4.1)	
9-12	26 (3.1)	11 (8.7)	37 (3.8)	
3-8	21 (2.5)	9 (7.1)	30 (3.1)	
**Vital Signs**				
HR (per min)	97.4±15.8	100.4±14.0	97.8±15.6	0.04
SBP (mmHg)	113.9±12.8	102.6±19.0	112.4±14.3	<0.0001
DBP (mmHg)	74.7±9.0	67.7±13.9	73.8±10.1	<0.0001
RR (per min)	13.7±1.8	16.3±2.6	14.0±2.2	<0.0001
SaO2 (%)	97.7±1.2	95.1±3.2	97.3±1.8	<0.0001
**Clinical findings**				
Dyspnea	72 (8.5)	78 (61.9)	160 (15.4)	<0.0001
Distracting pain	285 (33.6)	101 (80.2)	386 (39.6)	<0.0001
Chest skin abrasion	123 (14.5)	36 (28.6)	159 (16.3)	<0.0001
Chest tenderness	88 (10.4)	86 (68.2)	174 (17.9)	<0.0001
Chest deformity	0 (0.0)	10 (7.9)	10 (1.0)	<0.0001
Crepitation	2 (0.2)	31 (24.6)	33 (3.4)	<0.0001
Decreased pulmonary sound	5 (0.6)	49 (38.9)	54 (5.5)	<0.0001
Chest wall pain	131 (15.5)	105 (83.3)	236 (24.3)	<0.0001
Subcutaneous emphysema	3 (0.4)	19 (15.1)	22 (2.3)	<0.0001

Data are presented as mean ± standard deviation or frequency (%). DBP: Diastolic blood pressure; GCS: Glasgow coma scale; HR: Hear rate; RR: Respiratory rate; SBP: Systolic blood pressure; Sao2: Saturation of Oxygen.

**Table 2:** Independent predictors of positive chest radiography in pediatric trauma patients

**Variable**	**Odds ratio**	**95% CI**	**P**
Male gender	3.1	1.2-7.8	0.02
Decreased SBP	1.1	1.003-1.2	0.03
Decreased DBP	1.1	1.02-1.2	0.008
Decreased RR	1.3	1.1-1.5	0.002
Decreased pulmonary sound	64.4	15.8-262.0	<0.0001
Loss of consciousness	2.3	1.3-4.3	0.006
Chest tenderness	4.3	1.8-10.1	0.001
Crepitation	170.3	25.4-1141.4	<0.0001
Chest wall pain	145.2	49.2-428.8	<0.0001
Dyspnea	13.5	5.4-33.8	<0.0001
Chest skin abrasion	15.2	5.5-42.0	<0.0001

CI: Confidence interval; DBP: Diastolic blood pressure; RR: Respiratory rate; SBP: Systolic blood pressure.

**Table 3:** Screening performance characteristics of thoracic injury rule out criteria in prediction of positive chest radiography findings in children with trauma

**Performance**	**Value**	**95% CI**
True positive	126	---
True negative	764	---
False positive	84	---
False negative	0	---
Sensitivity	100.0	96.3-100.0
Specificity	90.1	87.8-92.0
Positive predictive value	60.0	53.0-66.6
Negative predictive value	100.0	99.4-100.0
Positive likelihood ratio	10.1	8.2-12.4
Negative predictive value	0.0	0.0-0.0

CI: Confidence interval.

**Figure 1:**
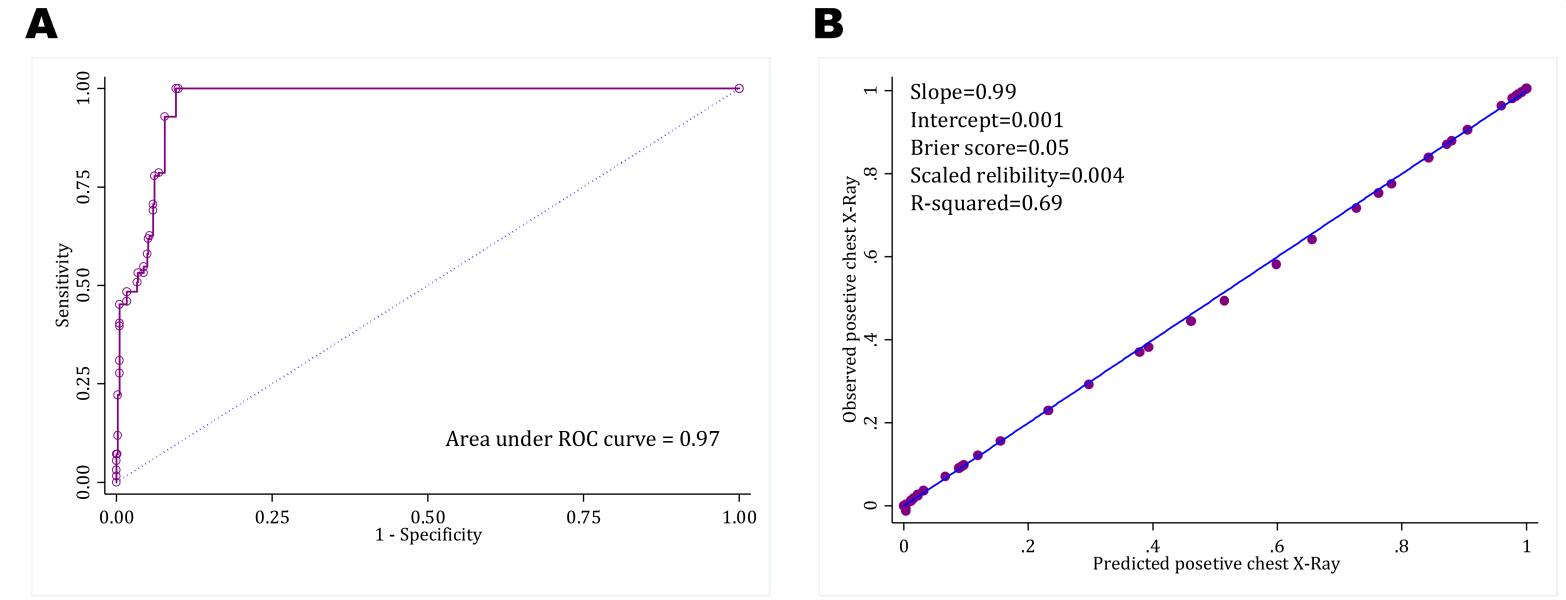
Area under the receiver operating characteristics (ROC) curve (A) and calibration plot (B) of thoracic injury rule out criteria in prediction of positive chest radiography findings in children with trauma.

**Figure 2:**
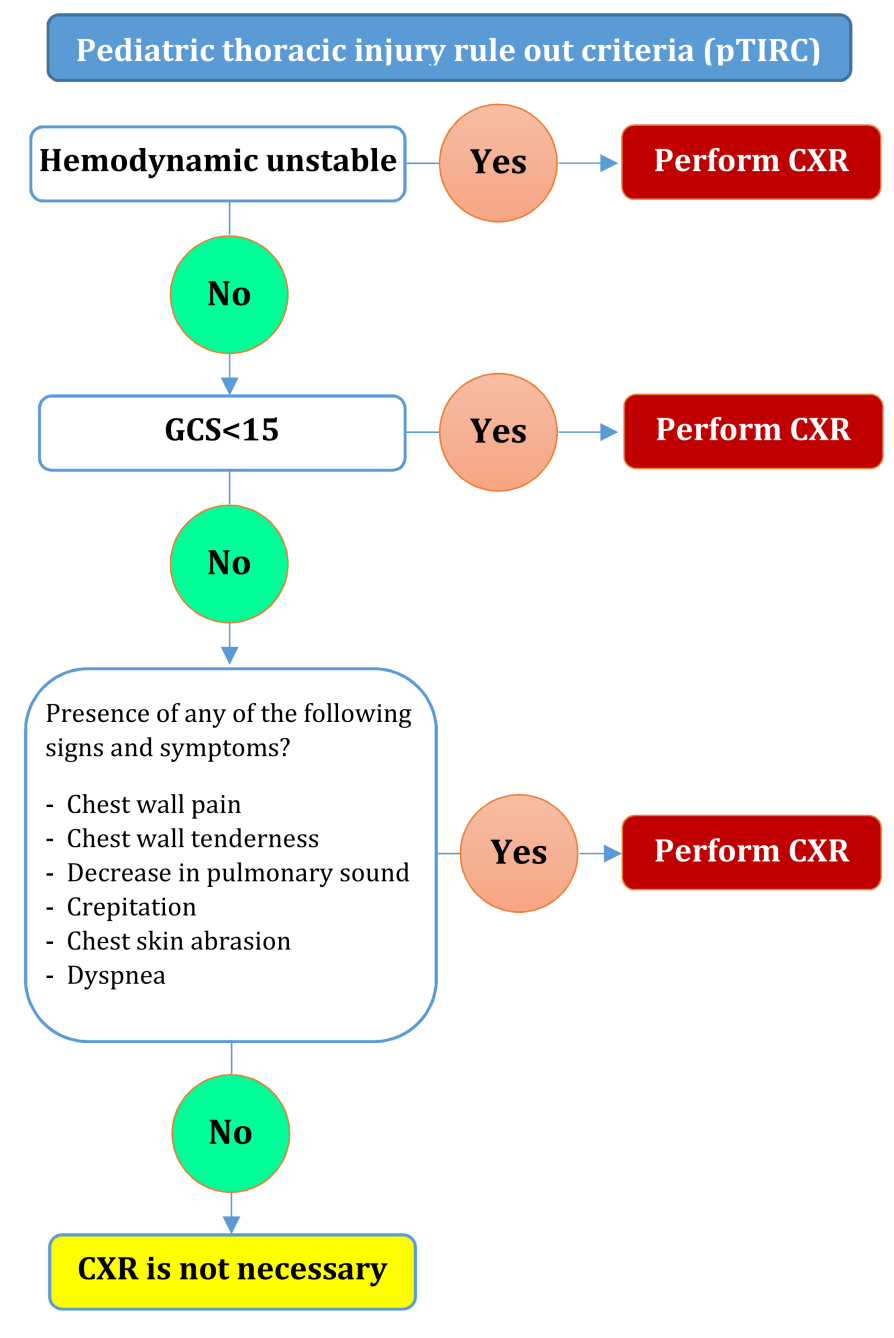
Pediatric thoracic injury rule out criteria (pTIRC) decision tree. CXR: chest X-Ray

Assessing blood pressure in children and adolescents was done according to previous studies (8-10). In summary, blood pressure was measured using an appropriate pediatric cuff from right arm based on krotocof sounds. Left hand was used if right hand was injured and a mercury sphygmomanometer was used in all health care centers. The chief researcher in each center confirmed the calibration of all sphygmomanometers as health care centers were using different brands.

Child Facial Coding System was used for assessing pain as it is challenging to interview and examine children to estimate the severity of their pain, especially in those less than 7 years of age (11). Therefore, facial expressions such as open lips, lowered brows, and mouth stretched wide in both directions were used to assess the presence or absence of pain in children under the age of 7. However, in children more than 7 years old, pain was assessed using interviews and examinations as this age group have higher cognitive skills. Level of consciousness was assessed using pediatric Glasgow coma scale (GCS) in children under 3 years (12), but standard GCS was used for children between the ages of 3 to 17 (13).

After clinical assessments, a CXR was obtained from children under study in antero-posterior and lateral views. Chest radiographs were interpreted by emergency medicine physicians in each center who were unaware of the clinical data. At the end, 5% of chest radiographs were presented to a radiologist to confirm the accuracy of the interpretations (inter-rater agreement= 98.3%).

Abnormal findings in the chest were identified using CXR. The presence of pneumothorax, hemothorax, lung contusion, pneumomediastinum, mediastinal widening, subcutaneous emphysema, fractures of ribs, sternum, clavicle and scapula were considered abnormal findings in chest radiographs.

***Statistical analysis***

Calculation of sample size was done using the method proposed in Hajian-Tilaki’s study (14). Therefore, a minimum sample size of 202 children was required considering a prevalence of 6.5% for the presence of abnormal findings in chest radiograph of patients with multiple trauma (15), area under the receiving operating characteristics (ROC) curve of 93% for TIRC in identification of abnormal findings in CXRs (16) and a marginal error of 3% (d=0.03) in estimation of the presence of an abnormality.

Data were analyzed using STATA version 14.0. Patients were categorized as two groups of CXR negative and CXR positive based on findings of chest radiographs and association between demographic factors, baseline factors and variables present in TIRC were assessed using chi^2 ^or t-test. Thereafter, all significant factors in univariate analysis were entered in a stepwise multiple logistic regression in order to identify independent predictors of positive CXR findings. Since variables from multivariate regression model were different from those of TIRC model designed by Forouzanfar et al. (6), some modifications were made to TIRC and pediatric TIRC (pTIRC) was introduced.

In the next step, calibration, discriminatory power and overall performance of this decision rule were assessed in order to evaluate the validity of pTIRC rule. Area under the ROC curve, sensitivity, specificity, positive predictive value, negative predictive value, and positive and negative likelihood ratio were calculated in order to assess the discriminatory power. In the present study, general calibration was assessed by drawing calibration plot. The closer the slope and intercept of calibration plot for pTIRC are to 1 and 0, respectively, the more perfect the model is for predicting the presence or absence of an abnormality in chest radiographs. In the end, overall performance was assessed using Brier score in order to assess the predictive accuracy and predictive reliability of the model.

## Results:

***Patients’ characteristics***

During the study period, data was gathered from 974 children consisting of 701 (72.0%) boys and 273 (28.0%) girls. These children had an average age of 8.8±4.3 years (1 to 17 years). Vehicle-pedestrian accident (38.9%), vehicle accident (28.7%) and motorcycle accidents (13.6%) were the most important mechanisms of injury. 869 (89.0%) children had a consciousness level of 15 based on GCS. Table 1 shows vital signs and clinical data of children on admission to emergency department. Chest radiograph findings were abnormal in 126 (12.9%) cases. Rib fracture (6.5%), lung contusion (6.4%), pneumothorax (4.4%) and hemothorax (4.0%) were the most common abnormal findings in chest radiographs.

***Predictors of positive CXR findings***

Most important predictors of positive CXR are shown in table 2. As shown, male gender (OR=3.1; p=0.02), decreased SBP (OR=1.1; p=0.03), decreased DBP (OR=1.1; p=0.008), decreased RR (OR=1.3; p=0.002), decreased pulmonary sound (OR=64.4; p<0.0001), loss of consciousness (OR=2.3; p=0.006), chest tenderness (OR=4.3; p=0.001), crepitation (OR=170.3; p<0.0001), chest pain (OR=145.2; p<0.0001), dyspnea (OR=13.5; p<0.001) and chest skin abrasion (OR=15.2; p<0.0001) were the most important predictors of positive CXR.

***Pediatric thoracic injury rule out criteria***

TIRC rule is designed for adults. As a result, age more than 60 years is included among factors affecting the prediction of positive CXR. In the present study, age was not a predicting factor of abnormal findings in chest radiograph of children and adolescents. Therefore, it was omitted from this model and Pediatric thoracic injury rule out criteria (pTIRC) was designed.

***Value of pTIRC rule in prediction of positive CXR in children***

**a) Discrimination**

Area under the ROC curve of pTIRC rule in prediction of abnormal CXR in children and adolescents was 0.97 (95% CI: 0.96-0.98) (figure 1A). Sensitivity and specificity of pTIRC were 100 and 90.1 percent, respectively. Positive and negative predictive values of pTIRC were 60.0 and 100 percent, respectively (table 3).

**b) Calibration**

The calibration plot of pTIRC rule in prediction of abnormal CXR findings in children and adolescents is depicted in figure 1B. As shown, calibration of pTIRC rule has great conformity with the ideal line with a slope of 0.99 and an intercept of 0.001.

**c) Overall performance**

In assessing overall performance, Brier score and scaled reliability of pTIRC in prediction of positive CXR findings were 0.05 and 0.004, respectively. Nagelkerke’s R^2 ^of this model was 0.69, which shows the good performance of pTIRC rule in prediction of abnormal findings in CXR of children and adolescents (figure 1B).

**Performance of pTIRC in decreasing unnecessary CXR**

The present study showed that chest radiographs were normal in 848 (87.1%) children and they were unnecessary. 764 (90.1%) of these unnecessary chest radiographs were identifiable using pTIRC. Therefore, there can be a 90.1% reduction in the number of unnecessary chest radiographs using pTIRC rule. No false negatives were observed in this decision rule. Decision tree of pTIRC rule is depicted in figure 2.

## Discussion:

Decreasing the number of unnecessary radiographs in chest traumas is an issue of great interest among researchers and there are many articles about this topic (6, 7, 15, 16). TIRC is one of the most important models introduced for predicting the presence of a positive CXR in adults (16, 17). In the present study, the value of this decision rule was assessed in children with omitting the age as a factor and then pTIRC was designed. Performance of pTIRC in prediction of positive CXR findings in children and adolescents with trauma is in an excellent level and has a sensitivity and specificity of 100 and 90.1 percent, respectively.

The present study is the first study assessing the value of clinical factors in predicting the presence of abnormal findings in CXR of children and adolescents in order to reduce the number of unnecessary radiographs. Therefore, the comparison of results of the present study with other studies is not feasible. 

TIRC was first designed and introduced by Forouzanfar et al. in 2014 (6). Another study by Safari et al. validated this model in adults (16). They showed that TIRC has a sensitivity and specificity of 100 and 67.7 percent, respectively, in patients with more than 14 years of age. Results from the present study in children between the ages of 1to 17 years are in accordance with the findings of previous studies.

In the fitted multivariable regression model of the present study, all variables present in TIRC, except age, had a meaningful association with the presence of positive CXR findings. Therefore, pTIRC was designed by omitting the age as a variable from the previous model and this omission did not influence the value of the mentioned model in prediction of positive findings in CXR of children. In addition, it seems that pTIRC in children has a greater value compared to TIRC in adults because the reported areas under the curve of studies by Frouzanfar et al. (AUC=0.94) and Safari et al. (AUC=0.93) were lower than area under the curve of the present study (AUC=0.97).

NEXUS chest is another model designed in the past few years in order to decrease the number of unnecessary chest radiographs (7). This model is also designed for adults and its value in children is not assessed yet. Additionally, there are other variables present in the NEXUS chest such as speed of the vehicle at the time of accident, the data of which are not accessible in many emergency wards, hence limiting the use of this decision rule in clinics, especially in developing countries. In contrast, variables present in pTIRC are readily accessible in clinics and this improves its applicability.

**Limitations**

Convenience sampling is one of the limitations of the present study. Continuous sampling could not be done as researchers were not present in emergency departments all day. Therefore, some degree of selection bias may be present in the study. Interpretation of chest radiographs by different physicians is another limitation of the present study. Finally, occult pneumothorax might have been present in some children who were categorized as healthy in the present study.

## Conclusion:

The present study assessed the value of TIRC in decreasing the number of unnecessary chest radiographs in traumatic children for the first time. The present study showed that among factors of TIRC rule, age does not have any association with Positive findings in CXR of children. Therefore, pTIRC was introduced after omission of age as a factor. pTIRC had an excellent performance in identification of traumatic chest injuries and decreasing the number of unnecessary chest radiographs.
